# Roles of DPY30 in the Proliferation and Motility of Gastric Cancer Cells

**DOI:** 10.1371/journal.pone.0131863

**Published:** 2015-07-06

**Authors:** Yong Joo Lee, Myoung-Eun Han, Su-Jin Baek, Seon-Young Kim, Sae-Ock Oh

**Affiliations:** 1 Departments of Anatomy, School of Medicine, Pusan National University, Busan, Republic of Korea; 2 Medical Research Center for Ischemic Tissue Regeneration, Pusan National University, Busan, Republic of Korea; 3 Medical Genomics Research Center, KRIBB, Daejeon, Republic of Korea; Wayne State University School of Medicine, UNITED STATES

## Abstract

Various types of histone methylation have been associated with cancer progression. Depending on the methylation site in histone proteins, its effects on transcription are different. DPY30 is a common member of SET1/MLL histone H3K4 methyltransferase complexes. However, its expression and roles in gastric cancer have been poorly characterized. To determine whether DPY30 has pathophysiological roles in gastric cancer, its expression and roles were examined. Immunohistochemistry and real time PCR showed up-regulation of DPY30 expression in some gastric cancer cell lines and patients’ tissues. Its knockdown by siRNA decreased the proliferation, migration, and invasion of gastric cancer cells, whereas its overexpression showed the opposite effects. These results indicate that DPY30 has critical roles in the proliferation, migration, and invasion of gastric cancer cells, and suggest DPY30 might be a therapeutic target in gastric cancer.

## Introduction

Covalent modifications of histone tails, such as, acetylation, phosphorylation, ubiquitination, and methylation, modulate chromatin structure and play pivotal roles in the regulation of cell-cycle progression, gene transcription, DNA repair, embryonic development, and cellular differentiation [1, 2]. While increased histone acetylation is generally associated with transcriptional activation, the methylation of histone is correlated with transcriptional activation and repression. For example, methylation of histone H3 at lysine 9, 20, or 27 residues (H3K9, H3K20 or H3K27) is involved in transcriptional gene silencing, but on the other hand, methylation at H3K4, H3K36, or H3K79 is associated with open chromatin and active gene transcription [3].

In mammals, mono-, di-, and tri-methylation of H3K4 (H3K4me1, H3K4me2, and H3K4me3) are performed by six distinct SET1/MLL family complexes (SET1A, SET1B, MLL1, MLL2, MLL3, and MLL4) [4, 5]. These H3K4 methyltransferases (H3K4MT) contain different catalytic subunits, and the activities of all six family complexes are controlled by common multi-subunit core components, which include WDR5, RBBP5, ASH2L, and DPY30, and are also referred to as WRADs. [6–9]. A loss of any subunit of WRAD complex results in reduced H3K4 methylation. WDR5 and RBBP5 are crucial for all three kinds of methylation of H3K4 (H3K4me1, H3K4me2, and H3K4me3), whereas ASH2L and DPY30 are mainly required for H3K4me3 [6, 8, 10, 11].

DPY30 is a member of all human SET1/MLL complexes, and is required for full SET1/MLL methyltransferase activity [10, 12]. DPY30 has been implicated in the differentiation potential of embryonic stem cells (ESCs) along the neuronal linage [10] and is essential for the proper differentiation and proliferation of hematopoietic progenitor cells [12]. Furthermore, depletion of DPY30 causes cells to enter a senescence-like state and to upregulate p16 (CDKN2A) and p15 (CDKN2B), which are directly involved in senescence [13].

Gastric cancer is one of the leading causes of cancer-related death worldwide [14]. Although recent diagnostic and therapeutic advances provide excellent survival for patients with early gastric cancer, the disease is usually diagnosed at a late stage when the prognosis is poor [15]. Gastric carcinogenesis entails the progressive accumulations of various genetic and epigenetic alterations, which lead to gain-of-function by oncogenes and loss-of-function by tumor suppressor genes. Furthermore, since gene transcription strongly relies on chromatin structure, altered or abnormal histone methylation has been typically associated with tumor progression and prognosis in cancer [16], and although the status of histone methylation has been well described in many types of cancer, this aspect remains unclear in gastric cancer [16, 17].

In this study, to determine whether DPY30 has pathophysiological roles in gastric cancer, its expression and roles were examined.

## Materials and Methods

### Cell culture and siRNA transfection

The gastric cancer-derived cell lines, SNU1, SNU16, SNU216, SNU620, SNU638, and NCI-N87 were purchased from the Korean Cell Line Bank (Seoul). The gastric epithelial cell line, HFE145 were gifted from Professor Hassan Ashktorab (Howard University). The gastric cancer cell lines were cultured at 37°C in a humidified 5% CO_2_/95% air atmosphere in RPMI1640 supplemented with 25 mM HEPES, 10% fetal bovine serum (FBS) (GE Healthcare Life Science, South Logan, UT, USA) and 100 μg/ml of penicillin/streptomycin (Sigma-Aldrich, St Louis, MO, USA). DMEM (GE Healthcare Life Science) medium supplemented with 10% FBS and 100 μg/ml of penicillin/streptomycin was used for HFE145 culture. Cells were transfected with small interfering RNA (siRNA) or scrambled (SCR) siRNA using DhamaFECT reagent 1 or 3 (Thermo Scientific, Lafayette, CO, USA) according to the manufacturer’s instructions. siRNA sequences were as follows: DPY30 siRNA duplex (ORF) (Bioneer, Daejeon, Korea), 5’-CAC UCU GAG UAC GGU CUC A(dTdT) -3’, 5’- CUC UGA GUA CGG UCU CAC A(dTdT) -3’, 5’- CUC ACU UAU UCU AGG UAC U(dTdT) -3’; DPY30 siRNA duplex (3’-UTR) (Bioneer); 5’- CCG GAC AAC AGA ACC UAU UUU UGG A(dTdT) -3’, 5’- GAG GCA GCU UUA AUU GCC AUG AUC A(dTdT) -3’; WDR5 siRNA duplex (Bioneer), 5’- GUC CUU GUG AAG CUC GUC U(dTdT) -3’, 5’- GUC GUG AUC UCA ACA GCU U(dTdT) -3’, 5’- CAG AUU CUA ACC UUC UUG U(dTdT) -3’; RBBP5 siRNA duplex (Bioneer), 5’- GUG UGA AAA GGG CUC AGU A(dTdT) -3’, 5’- CAG AUU CUC AGG AUC UUG U(dTdT) -3’, 5’- GUG GUU GAG AUU AGU AGA U(dTdT) -3’; ASH2L siRNA duplex (Bioneer), 5’- GUA UGA ACG GGU UUU GUU A(dTdT) -3’, 5’- CUG AGA ACA CCU GAA AUC A(dTdT) -3’, 5’- GUC UAC CUU UCA UGA CCA A(dTdT) -3’; scrambled (SCR) siRNA (Thermo Scientific), 5’- GAU CCG CAA AAG AGC GAA A(dTdT) -3’.

### DPY30 overexpression

The DPY30 cDNA was cloned into pLenti6.3-V5/DEST vector (lentiviral destination vector) using the *in vivo* recombination-based Gateway cloning system (Invitrogen, Carlsbad, CA, USA). The donor vector (pDONR221) harboring the cDNA sequence of DPY30 was purchased from Ultimate ORF Clones (Invitrogen), and recombined with the counter-selectable *ccdB* gene of pLenti6.3-V5/DEST using LR clonase enzyme mixture (Invitrogen). The empty vector pLenti6.3/V5-DEST was used as a mock control. Recombinant lentiviruses were produced in 293FT cells, and used to infect SNU216 and SNU638 cells, according to the manufacturer’s instructions (ViraPower Lentiviral Expression System; Invitrogen). DPY30-overexpressing stable cells were established by selection with blasticidin (7.5 μg/ml) (Invitrogen).

For rescue assays, we modified, followed a protocol ‘RNAi interference using Precision Lenti ORF collection’ (http://dharmacon.gelifesciences.com/uploadedFiles/Resources/precision-lentiorfs-rnai-rescue-appnote.pdf). Briefly, the cells were infected with recombinant lentiviruses (pLenti6.3-V5/DEST or DPY30-over constructs). 24 hours post-transduction, the media containing virus were removed and replaced with complete culture medium. The following day, blasticidin was added at a concentration of 7.5 μg/ml. Cells were maintained under selection for six days, replacing medium or passaging every 2–3 days as needed. The selected populations of control cells or cells expressing DPY30 were used for transfection experiments using DPY30 siRNA targeting the open reading frame (ORF) or 3’-untranslated region (UTR).

### Real-time PCR

Tissue samples were obtained from 23 unrelated Korean primary gastric cancer patients that underwent surgical resection at Pusan National University Hospital and Pusan National University Yangsan Hospital and provided written informed consent. The study was approved by the Pusan National University Hospital-Institutional Review Board (PNUH-IRB) and the Pusan National University Yangsan Hospital-Institutional Review Board (PNUYH-IRB). Total RNA from tissues and cells were extracted using Trizol reagent (Invitrogen) or an RNeasy Mini kit (Qiagen, Valencia, CA, USA), according to the manufacturers' instructions. Quantitative real-time polymerase chain reaction (real-time PCR) was used to check DPY30 mRNA levels. cDNAs were synthesized with MMLV reverse transcriptase (Promega, Madison, WI, USA), dNTP, and oligo-dT primers. The primer sequences used were as follows: DPY30, 5’- AAC GCA GGT TGC AGA AAA TCC T -3’ and 5’- TCT GAT CCA GGT AGG CAC GAG -3’; WDR5, 5’- TGT TAC TGG TGG GAA GTG GA -3 and 5’- CTG TTG GGT GAC AAG CTG TT -3’; RBBP5, 5’- AGT GCA CAC ATC CAT CCA GT -3’ and 5’- TCA CAG TCG CCT GAA AGA AC -3’; ASH2L, 5’- TAC AAG AGC TGC ACG GTT TC -3’ and 5’- CCA GCC CAT GTC ACT CAT AG -3’; GAPDH, 5’- TGG GCC AGG AAA TCA CAT CC -3’ and 5’- CTC AGC CCG AGT GGA AAT GG -3’. Real-time PCR was performed using a LightCycler™ 96 Real-time PCR system (Roche, Nutley, NJ) and FastStart Essential DNA Green Master (Roche), according to the manufacturer's instructions. GAPDH was used as an internal control.

### Immunohistochemistry

Immunohistochemical staining with rabbit anti-human DPY30 polyclonal antibody (Sigma-Aldrich) was performed on a gastric cancer tissue array (n = 59) purchased from SUPER BIO CHIPS (Seoul) or on 4-μm sections of paraffin-embedded specimens. Briefly, after deparaffinization and rehydration, slides were treated with 0.3% hydrogen peroxide for 30 min to inhibit endogenous peroxidase activity, and blocked with 10% normal donkey serum (NDS) and 1% BSA in 1×phosphate buffered saline (PBS). Slides were then incubated overnight at 4°C in blocking buffer containing a rabbit anti-human DPY30 primary antibody (1:80, Sigma-Aldrich). Secondary antibody (HRP-conjugated) binding was performed at a dilution of 1:200 in blocking buffer for 2h at RT. Detection was performed with HRP (Vector Laboratories), and slides were counterstained by treating them for 1 min with hematoxylin staining buffer (Sigma-Aldrich).

### Cell proliferation assay

One day after transfecting cells with siRNA, the medium was replaced with 1% FBS medium, and four days later, 10 μl of Ez-Cytox (ITSBIO, Seoul) was added and incubated for 0.5 to 2 hrs. In order to check the effect of DPY30 overexpression on cell proliferation, cells were seeded in 10% FBS medium, and after three days of culture, 10 μl of Ez-Cytox (ITSBIO) was added and incubated for 0.5 to 2 hrs. Cell viabilities were determined by measuring absorbance at 450 nm using VICTOR3 multiple readers (PerkinElmer, MA, USA).

### Boyden chamber assay

A modified Boyden transwell chamber (Neuro Probe, Gaithersburg, MD, USA) was used. The bottom chamber was filled with 50 μl RPMI containing 10% FBS. To examine effects of DPY30 known-down, SNU1 and SNU16 (non-adherent cells) were transfected with the ORF-targeting siRNA. One day after transfection, cells were washed, suspended at a density of 5 × 10^5^ cells/ml in 50 μl of RPMI supplemented with 0.5% FBS, and seeded into the upper chamber. To remove the effects of proliferation, mitomycin C (0.01 μg/ml, Sigma-Aldrich) was added. The cells were then incubated for 24 hrs at 37°C with 5% CO2. The number of migrated cells was evaluated by counting cells in bottom wells. To examine effects of DPY30 overexpression, the stable cells (adherent cells; SNU216 and SNU638) were used. Cells (mock and DPY30-over) were trypsinized, and seeded at a density of 1 × 10^5^ cells/ml. To remove the effects of proliferation, mitomycin C was added. Cells were allowed to migrate for four hrs, membranes were fixed and stained using Diff-quik solution (Sysmex, Kobe, Japan) and washed with distilled water. Cell numbers in 10 randomly chosen fields were counted using a light microscope. Experiments were performed in triplicate.

### Matrigel invasion assay

The invasive abilities of cancer cells were assessed using 8-μm porous BioCoat Matrigel chamber inserts (BD Bioscience, San Jose, CA, USA). One day after transfection with SCR or DPY30 siRNA, cells were trypsinized, and suspended at a density of 5 × 10^4^ cells/ml in 500 μl of RPMI supplemented with 0.5% FBS and mitomycin C (0.01 μg/ml, Sigma-Aldrich). Cells were then added to chamber inserts and placed in wells filled with 0.7 ml of medium supplemented with 10% FBS as chemoattractant. After incubation for 24 or 48 hrs, non-invading cells on top of the membrane were removed by scraping, and invasive cells on the bottom of the membrane were fixed and stained with Diff-quik solution (Sysmex). Cell numbers in 10 randomly chosen fields were counted using a light microscope. Experiments were performed in triplicate and at least 5 fields were counted per experiment.

### Statistical analysis

Results are expressed as the means ± standard deviations (SDs) of three independent experiments. The significances of differences were determined using the Student’s *t*-test for unpaired observations. *P* values of < 0.05 were considered statistically significant.

## Results

### Expression of DPY30 in gastric cancer tissues

**Fig 1:**
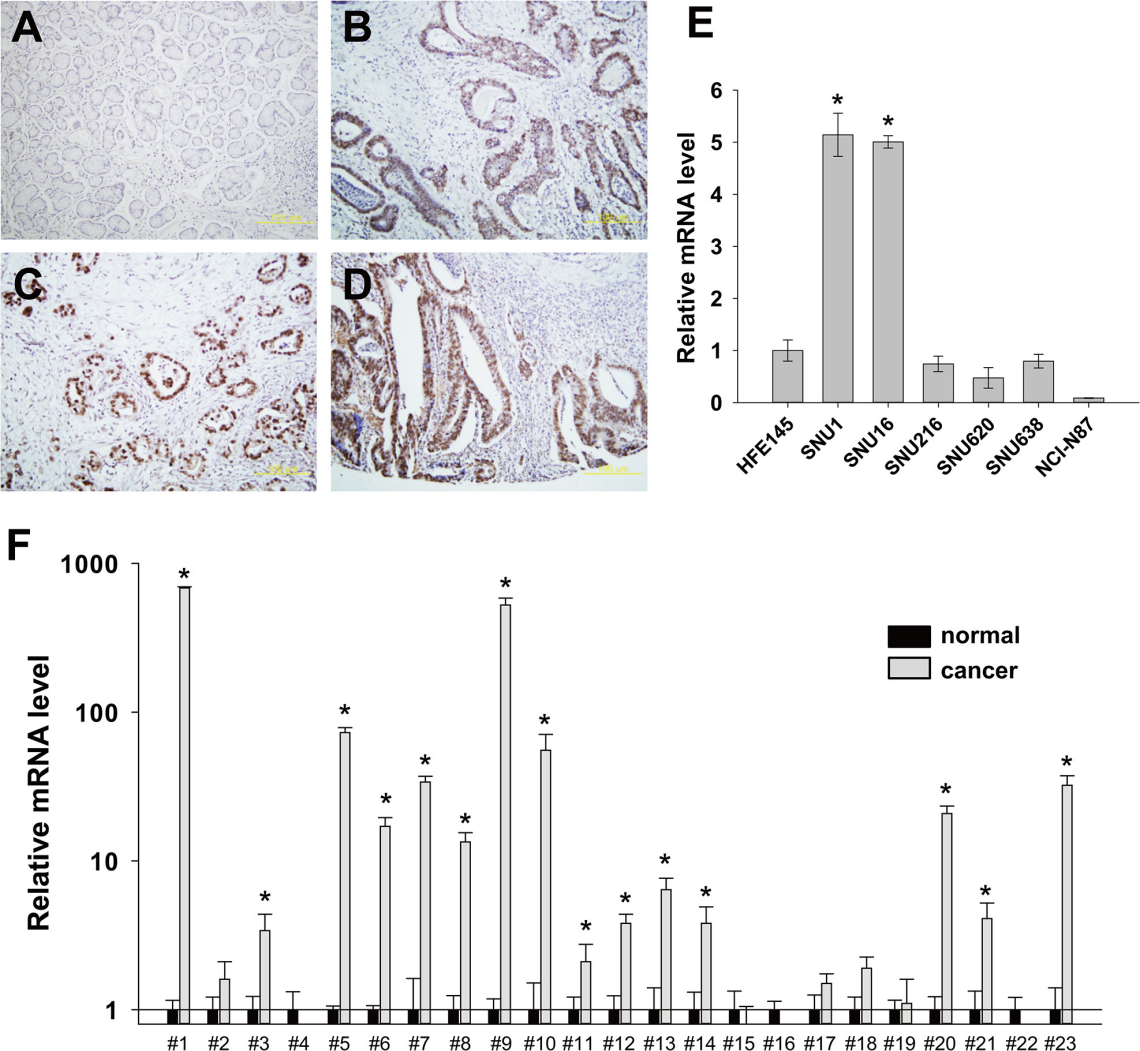
Overexpression of DPY30 in gastric cancers. (A-D) Immunohistochemical staining demonstrated the overexpression of DPY30 in gastric cancer tissues. Notably its overexpression was obviously greater in invading cancer cells (B-D) than in normal gastric mucosa (A). (E) The mRNA level of DPY30 in gastric cancer cells (SNU1, SNU16, SNU216, SNU620, SNU638 and NCI-N87) and normal gastric epithelial cell (HFE145) was determined by real-time PCR using specific primers for DPY30. GAPDH was used to normalize data. Values shown are the means ± SDs of the three independent experiments performed in triplicate. *, *p* < 0.01 (Student’s *t* test, versus HFE145). (F) The expression of DPY30 in gastric cancer tissues was examined by real-time PCR using specific primers for DPY30. GAPDH was used to normalize data. Values are the means ± SDs of three independent experiments performed in triplicate. *, *p* < 0.01; **, *p* < 0.05 (Student’s *t* test, versus normal).

To investigate DPY30 expression in human gastric cancer, we performed immunohistochemistry using a gastric cancer tissue array or archival paraffin-embedded tissue sections. In normal gastric tissues, DPY30 expression was difficult to detect (Fig 1A), but in cancer tissues DPY30 protein was widely overexpressed (Fig 1B–1D). Notably, DPY30 was overexpressed in invading gastric cancer cells (Fig 1B–1D). Furthermore, we determined mRNA levels of DPY30 in one immortalized normal gastric epithelial cell line (HFE145) and six gastric cancer-derived cell lines (SNU1, SNU16, SNU216, SNU620, SNU638 and NCI-N87) using real-time PCR. The mRNA level of DPY30 was considerably higher (fold change > 5) in SNU1 and SNU16 than in HFE145 cells, whereas the expression level of DPY30 in SNU216, SNU620 and SNU638 was similar to those in HFE145 cells and was lower (fold change > 10) in NCI-N87 (Fig 1E). We also checked mRNA levels of DPY30 in gastric cancer tissues. DPY30 was highly expressed in 15 cases (15/23, 65%) as compared with normal tissues (Fig 1F). These results indicate that DPY30 is highly expressed in some human gastric cancers.

### Roles of DPY30 in the proliferation of gastric cancer cells

**Fig 2:**
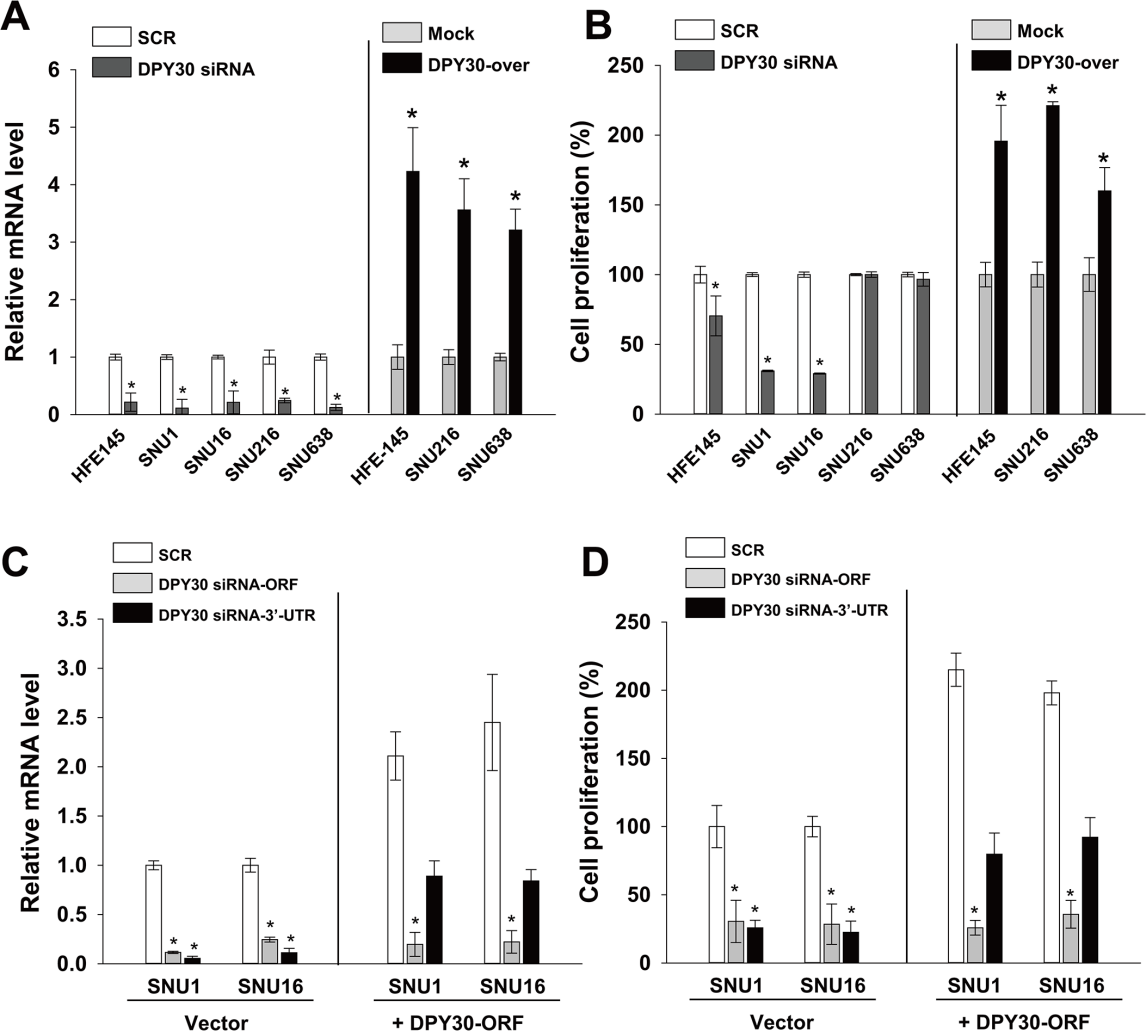
DPY30 regulated the proliferation of gastric cancer cells. (A) Real-time PCR was used to determine the efficiency of knockdown or overexpression of DPY30 in HFE145, SNU1, SNU16, SNU216 and SNU638 cells. Knockdown efficiency was determined after transfecting cells with 100 nM DPY30 siRNA targeting the ORF or scrambled siRNA (SCR). Overexpression efficiencies were determined in DPY30-overexpressing and mock cells. (B) Effect of DPY30 knockdown or overexpression on cell proliferation. A cell viability assay was used to measure cell proliferation in the presence of 1% FBS. For the DPY30 knockdown experiments, cell viability assays were performed five days after transfecting 100 nM DPY30 siRNA or SCR. After three days of culture, cell viability assays were performed on DPY30-over and mock cells. (C) Expression analysis of DPY30 after known-down or overexpression by siRNAs or DPY30 ORF respectively. The ORF-targeting or the 3’-UTR-targeting siRNA was used for the knock-down. (D) Exogenous DPY30-ORF rescued the inhibition of proliferation by the 3’-UTR-targeting DPY30 siRNA. Mock or DPY30-overexpressing cells were transduced with two kinds of DPY30 siRNA (3’-UTR-targeting or ORF-targeting), then cell viability assay was examined. Values are the means ± SDs of three independent experiments performed in triplicate. *, *p* < 0.01 (Student’s *t* test, versus SCR or Mock).

In order to determine possible roles of DPY30 in gastric cancer cells, we first knocked-down DPY30 using the ORF-targeting DPY30 siRNA and monitored its knockdown efficiency by real-time PCR (Fig 2A). The ORF-targeting DPY30 siRNA (100 nM) decreased the mRNA level of DPY30 in HFE145, SNU1, SNU16, SNU216, and SNU638 cells as compared with scrambled siRNA (SCR) by 78%, 89%, 79%, 76%, and 88%, respectively. Five days after transfecting cells with SCR or the ORF-targeting siRNA, we performed proliferation assays. Knockdown of DPY30 inhibited the proliferations of HFE145, SNU1 and SNU16 as compared with SCR by 31%, 69% and 71% respectively, while the knockdown did not affect the proliferations of SNU216 and SNU638 (Fig 2B), in which the expression level of DPY30 was low (Fig 1E). Next, we produced DPY30-overexpressing cell lines (DPY30-over) using HFE145, SNU216 and SNU638 cells, and then compared DPY30 mRNA levels in mock cells (control) and DPY30-over cells. Real-time PCR results showed that the expression level of DPY30 was 4.2, 3.5 and 3.2 fold higher in DPY30-overexpressing HFE145, SNU216 and SNU638 cells than mock cells, respectively (Fig 2A). DPY30 overexpression enhanced the proliferations of HFE145, SNU216 and SNU638 cells versus mock cells by 1.9, 2.2 and 1.6 fold, respectively (Fig 2B).

To confirm the specificity of DPY30 siRNA, we performed the rescue experiments. For the rescue experiments, we used a DPY30 siRNA targeting 3’-UTR instead of ORF because the ORF-targeting siRNA can also degrade the exogenous DPY30 ORF DNA we introduced. To overexpress DPY30, we transduced SNU1 and SNU16 with viral particles, pLenti6.3-V5/DEST (control) or DPY30-ORF-over constructs. DPY30-overexpressing SNU1 or SNU16 cells showed higher expression of DPY30 2.1 or 2.5 fold higher than mock cells, respectively (Fig 2C). The ORF-targeting siRNA led to down-regulation of DPY30 expression levels by greater than 75% in mock as well as DPY30-overexpressing cells. The 3’-UTR-targeting siRNA down-regulated the DPY30 expression by greater than 85% in mock cells, whereas, in the DPY30-overexpressing cells, it decreased the DPY30 expression to a level similar to that of SCR siRNA-transfected cells (Fig 2C). This result indicates that the exogenous DPY30-ORF DNA is resistant to the 3’-UTR-targeting siRNA and is expressed at a similar level to that of endogenous DPY30 mRNA. The ORF-targeting siRNA inhibited proliferation of cells regardless of the exogenous DPY30 expression (Fig 2D). Proliferation was also inhibited by the 3’UTR-targeting siRNA in mock cells, however it was partially inhibited in DPY30-overexpressing cells. In DPY30-overexpressing cells, the ORF-targeting siRNA decreased the proliferation of SNU1 and SNU16 as compared with SCR siRNA by 74% and 66%, respectively, however the 3’-UTR-targeting siRNA decreased by 20% and 8%, respectively. This result indicates that exogenous DPY30 rescues the inhibition of proliferation induced by the 3’-UTR-targeting siRNA, and the phenotypes caused by DPY30 specific siRNA are not off-target effects.

**Fig 3:**
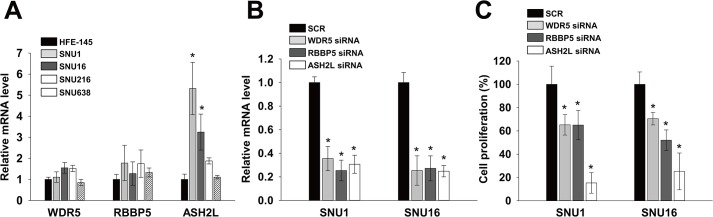
WRAD components regulated proliferation of gastric cancer cells. (A) The mRNA levels of WDR5, RBBP5 and ASH2L in HFE145, SNU1, SNU16, SNU216 and SNU638 cells were determined by real-time PCR, using specific primers for WDR5, RBBP5 and ASH2L. GAPDH was used to normalize data. (B) Knockdown efficiency was determined by real-time PCR. Knockdown efficiency was determined after transfecting cells with 100 nM WDR5, RBBP5 and ASH2L siRNA or scrambled (SCR). (C) Effects of WDR5, RBBP5 and ASH2L knockdown on cell proliferation. A cell viability assay was used to measure cell proliferation in the presence of 1% FBS. 24 hrs after transduction, cell viability assays were performed. Values are the means ± SDs of three independent experiments performed in triplicate. *, *p* < 0.01 (Student’s *t* test, versus HFE145 (A) or SCR (B-C)).

Because DPY30 is a component of WARDs, we examined expressions and roles of other components of WARDs. Real-time PCR showed that mRNA levels of WDR5 and RBBP5 in SNU1, SNU16, SNU216 and SNU638 were similar to those of HFE145. However, the mRNA level of ASH2L were significantly higher (fold change > 3) in SNU1 and SNU16 than in HFE145 cells (Fig 3A) as DPY30 in Fig 1E. In order to investigate whether WRAD components are associated with proliferation of gastric cancer cells, we knockdowned each WRAD component using their specific siRNA and monitored knockdown efficiencies by real-time PCR (Fig 3B). Each siRNA (100 nM) decreased the mRNA levels in SNU1 and SNU16 cells as compared with SCR by 65%-75%. Knockdown of WDR5 and RBBP5 inhibited the proliferation of SNU1 and SNU16 cells as compared with SCR by 30–48% (Fig 3C). However, ASH2L inhibited the proliferation of SNU1 and SNU16, in which the expression level of ASH2L was high, as compared with SCR by 85% and 75%, respectively.

### Roles of DPY30 in the migration and invasion of gastric cancer cells

**Fig 4:**
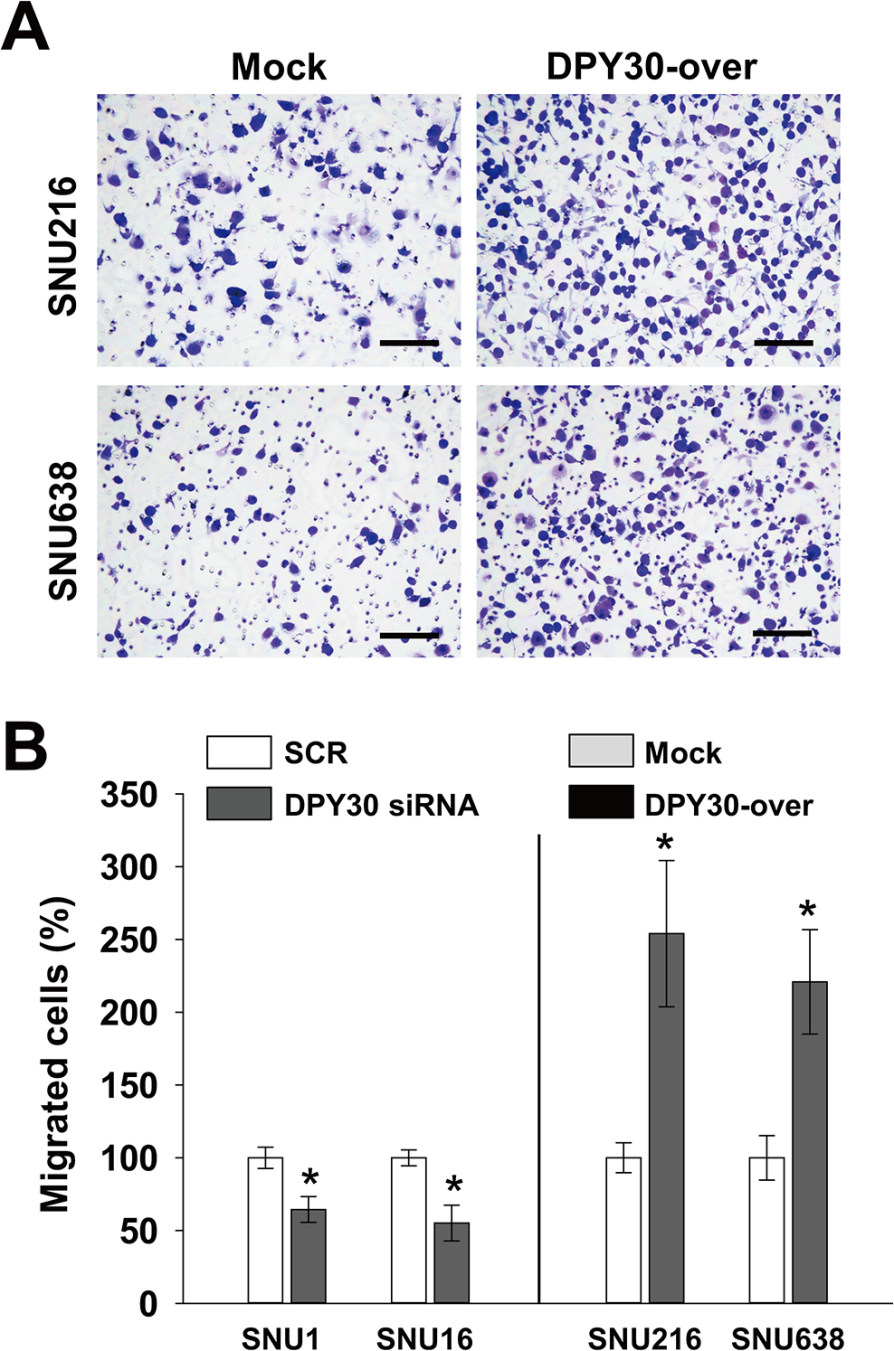
DPY30 regulated the migration of gastric cancer cells. (A) A Boyden chamber assay was used to measure the migration of gastric cancer cells. 10% FBS was used to induce migration, and mitomycin C (0.01 μg/ml) was added to remove the effects of proliferation. Two days after transfection with 100 nM ORF-targeting DPY30 siRNA or scrambled (SCR) siRNA, migration assays (Boyden chamber assay) were performed. Migration assays were carried out on DPY30-over and mock cells after culture for one day. Bar = 100 μm. (B) Migrated cells were counted and results are presented as a bar graph. Values are the means ± SDs of three independent experiments performed in triplicate. *, *p* < 0.01 (Student’s *t* test, versus SCR or Mock).

**Fig 5:**
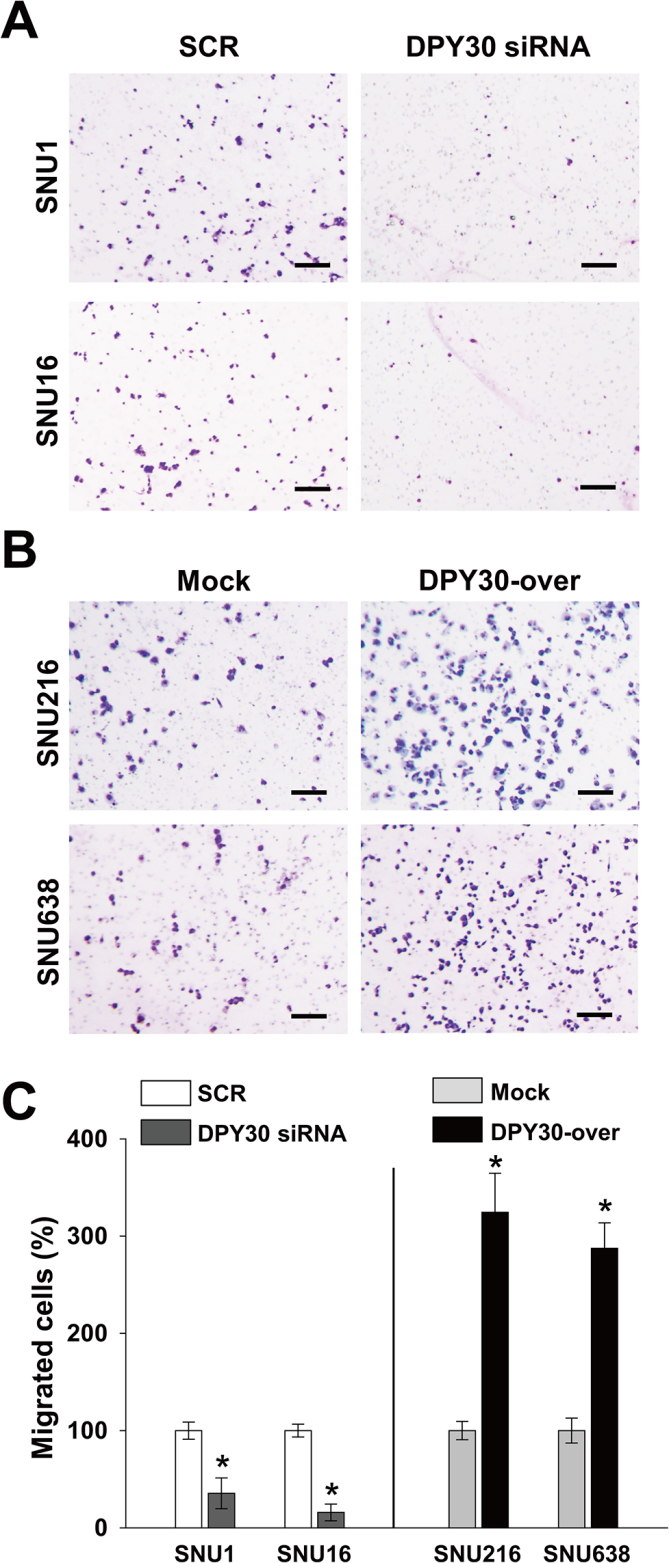
DPY30 regulated the invasion of gastric cancer cells. (A-B) A Matrigel invasion assay was used to measure gastric cancer cell invasion. Presented results are representative of the results obtained. 10% FBS was used to induce invasion, and mitomycin C (0.01 μg/ml) was added to remove effects of proliferation. Invasion assays were performed two days after transfection with 100 nM ORF-targeting DPY30 or SCR siRNA. After culture for one day, invasion assay was carried out for the DPY30-over and mock cells. Bar = 100 μm. (C) Invasive cells were counted and results are displayed as a bar graph. Values are the means ± SDs of three independent experiments performed in triplicate. *, *p* < 0.01 (Student’s *t* test, versus SCR or Mock).

We next examined whether DPY30 regulates the migration of gastric cancer cells. Knockdown of DPY30 decreased the FBS-induced migrations of SNU1 and SNU16 cells versus SCR by 35% and 45%, respectively (Fig 4). In contrast, DPY30 overexpression increased the FBS-induced migrations of SNU216 and SNU638 cells versus mock cells by 2.5 and 2.2 fold, respectively (Fig 4). These results led us to examine the role of DPY30 in the invasion of gastric cancer cells. In a Matrigel invasion assay, DPY30 siRNA inhibited the FBS-induced invasions of SNU1 and SNU16 cells versus SCR siRNA by 65% and 84%, respectively (Fig 5A and 5C). Furthermore, DPY30 overexpression increased the FBS-induced invasions of SNU216 and SNU638 cells versus mock cells by 3.2 and 2.9 fold, respectively (Fig 5B and 5C). These results show that DPY30 promotes the migration and invasion of gastric cancer cells.

## Discussion

Roles of DPY30 in cancer biology have been poorly characterized although its roles in differentiation of ESCs and hematopoietic progenitor cells had been reported [10, 12]. In the present study, we showed novel roles of DPY30 in gastric cancer. DPY30 is amplified (data not shown) and highly expressed (Fig 1) in some gastric cancer tissues. Moreover, its knockdown or overexpression regulated the proliferation, migration and invasion of gastric cancer cells. Alterations in the expression, chromosomal translocations and somatic mutations of genes encoding subunits of SET1/MLL complexes have been reported in many cancers. These results support the critical roles of members of SET1/MLL complex in cancer progression.

Up-regulation of DPY30 expression was observed only in SNU1 and SNU16 gastric cancer cells among six gastric cancer cells we have examined. However its up-regulation was observed in more than half of gastric cancer tissues we have examined (Fig 1). It is hard to explain this discrepancy. However, it is obvious that the number of gastric cancer tissues we have examined is not enough. So, an extensive expression study using much more number of tissues is required to evaluate the clinical value in the future.

Roles of SET1/MLL H3K4 methyltransferase complex in cancer biology is complex. Regulatory subunits of SET1/MLL complexes, including WRAD, contain both potent oncoproteins and tumor suppressors. For example, the MEN1 gene encodes menin, an integral subunit of MLL1 and MLL2, and is mutated in patients with multiple endocrine neoplasia type 1 (MEN1) [18, 19]. Furthermore, menin regulates p18 (CDKN2C) and p27 (CDKN1B) by recruiting MLL1 complex [20]. In contrast, WDR5, a component of WRAD complex, has been reported to act as an oncoprotein in prostate cancer, in which it is overexpressed and crucial for the androgen-induced proliferation of tumor cells [21]. ASH2L, which is another component of WRAD complex and can interact with the oncoprotein MYC [22], is crucial for the MYC/Ha-RAS-dependent transformation of rat embryo fibroblasts. Moreover, it is overexpressed in many kinds of tumors and is critical for the proliferation of tumor cells [23]. In the present study, we showed roles of DPY30 as an oncoprotein in the gastric cancer.

What are molecular mechanisms underlying roles of DPY30 in gastric cancer? Although we did not reveal, several hypotheses can be proposed based on previous studies. One of possible hypotheses is that DPY30 overexpression leads to the overexpressions of oncogenes via increased H3K4MT activity. Depletion of DPY30 or ASH2L leads to a decrease in H3K4me3, while WDR5 and RBBP5 are crucial for all three kinds of H3K4 (H3K4me1, H3K4me2, and H3K4me3) [6, 8, 10, 11]. In addition, the overexpression of DPY30 alone can increase H3K4MT activity [10]. Since H3K4me2/3 is an active mark for transcription [3, 24], increased H3K4MT may directly upregulate many genes, such as, oncogenes, or alternatively indirectly down-regulate tumor suppressors. One previous report supporting this hypothesis is that ASH2L, a critical component of SET1/MLL complex, has been shown to play as an oncoprotein [25, 26]. Another previous report is that DPY30 directly activates the expressions of ID proteins via H3K4 methylation [13, 27]. ID proteins inhibited activities of ETS1 and ETS2 which can induce one of tumor suppressor gene, p16. In the present study, we showed that components of WRAD (WDR5, RBBP5 and ASH2L) can inhibit proliferation of gastric cancer cells (Fig 3), suggesting that DPY30 act through the increased H3K4MT activity. More studies are required to reveal the mechanisms underlying the roles of DPY30 in gastric cancer cells.
